# Direct purification and immobilization of his-tagged enzymes using unmodified nickel ferrite NiFe_2_O_4_ magnetic nanoparticles

**DOI:** 10.1038/s41598-023-48795-x

**Published:** 2023-12-06

**Authors:** Elizabeth C. H. T. Lau, Kimberley C. Dodds, Catherine McKenna, Rhona M. Cowan, Alexey Y. Ganin, Dominic J. Campopiano, Humphrey H. P. Yiu

**Affiliations:** 1 Chemical Engineering, School of Engineering and Physical Sciences, Heriot-Watt University, Edinburgh, EH14 4AS UK; 2 School of Chemistry, University of Edinburgh, Edinburgh, EH9 3FJ UK; 3 School of Chemistry, University of Glasgow, Glasgow, G12 8QQ UK

**Keywords:** Nanobiotechnology, Nanoscale materials, Nanoscience and technology

## Abstract

Purification of valuable engineered proteins and enzymes can be laborious, costly, and generating large amount of chemical waste. Whilst enzyme immobilization can enhance recycling and reuse of enzymes, conventional methods for immobilizing engineered enzymes from purified samples are also inefficient with multiple-step protocols, regarding both the carrier preparation and enzyme binding. Nickel ferrite magnetic nanoparticles (NiFe_2_O_4_ MNPs) offer distinct advantages in both purification and immobilization of enzymes. In this work, we demonstrate the preparation of NiFe_2_O_4_ MNPs via a one-step solvothermal synthesis and their use in direct enzyme binding from cell lysates. These NiFe_2_O_4_ MNPs have showed an average diameter of 8.9 ± 1.7 nm from TEM analysis and a magnetization at saturation (M_s_) value of 53.0 emu g^–1^ from SQUID measurement. The nickel binding sites of the MNP surface allow direct binding of three his-tagged enzymes, d-phenylglycine aminotransferase (d-PhgAT), *Halomonas elongata* ω-transaminase (HeωT), and glucose dehydrogenase from *Bacillus subtilis* (*Bs*GDH). It was found that the enzymatic activities of all immobilized samples directly prepared from cell lysates were comparable to those prepared from the conventional immobilization method using purified enzymes. Remarkably, d-PhgAT supported on NiFe_2_O_4_ MNPs also showed similar activity to the purified free enzyme. By comparing on both carrier preparation and enzyme immobilization protocols, use of NiFe_2_O_4_ MNPs for direct enzyme immobilization from cell lysate can significantly reduce the number of steps, time, and use of chemicals. Therefore, NiFe_2_O_4_ MNPs can offer considerable advantages for use in both enzyme immobilization and protein purification in pharmaceutical and other chemical industries.

## Introduction

Protein purification is often employed to isolate a single type of protein from a complex biomolecular mixture, e.g. from animal or plant tissues, or from cell lysate^1–4^. However, it can involve many separation techniques and steps to attain the desired protein^5,6^. As a result, a substantial amount of time and chemicals/materials (e.g. surfactants) are consumed in order to successfully purify the required protein from the starting material^5,7^ significantly increasing the operational cost^6^. Indeed, the pharmaceutical and biotechnological sectors are considered to be less environmentally friendly (measured with “E-factor”) than petroleum industries regarding waste emissions^8^. Under the current climate regarding sustainability, these sectors need to reduce their waste emissions but retain productivity. Simplifying the purification protocols of biomolecules, including proteins and enzymes, would help to reduce waste emissions significantly.

In order to facilitate a reduction in steps and chemical usage for protein purification, a histidine peptide chain, or “his-tag”, was added to the recombinant protein expressions via genetic engineering^9–12^. His-tagged proteins and enzymes can be purified using immobilized-metal affinity chromatography (IMAC), which exploits the strong co-ordinate bonds formed the his-tag and the transition metal ions in the support (or stationary phase), using Ni columns to trap the desired protein^13–16^. Nickel, notably Ni–NTA (Ni^2+^ ions coupled to nitrilotriacetic acid), has become the most commonly used metal ion species in IMAC due to its high binding affinity to his-tag at pH 8 or higher^9,17^. Eluting for the bound his-tagged proteins can be carried out by reducing the pH or using competitive, strong chelating agents such as EDTA or imidazole, which would compete with the his-tags for the Ni sites^18–21^. In general, adding a his-tag on to a protein can simplify the purification and allow for a high purity sample^22^. Exploiting such use of his-tag chelation to metal ions can enhance purification and immobilization of proteins in one step onto a carrier with accessible nickel sites.

Scientists have also been exploiting the binding property of his-tags to nickel for enzyme immobilization^23–27^. For example, Zhou et al. reported the use of a non-magnetic carrier, Ni–NTA on mesoporous silica nanoparticles, to immobilize his-tagged organophosphohydrolase from *Agrobacterium radiobacter* P230 (OpdA) for the bioremediation of organophosphates in soil^28^. Although this Ni-loaded carrier allowed an in tandem purification and immobilization of enzymes directly from the cell lysate, the synthesis of carrier was rather complicated with a protocol of at least five steps, including the synthesis of mesoporous silica nanoparticles and grafting Ni–NTA, which may not enhance sustainability and cost effectiveness^29^. Magnetic supports with Ni binding sites can add further advantages in separation if the matrices contain considerable amount of unwanted solid such as debris from cell cultures. In the literature, one-pot purification and immobilization of enzymes has also been reported using magnetic materials functionalized with Ni–NTA functional groups^30,31^. For example, Wang et al. synthesized poly(glycidyl methacrylate) (PGMA) coated Fe_3_O_4_ MNPs with Ni–NTA as the nickel source for chelate a his-tagged enzyme epoxide hydrolase directly from a cell lysate^30^. However, the synthesis of these particles involves at least six steps from the preparation of oleic acid coated Fe_3_O_4_ MNPs to the Ni–NTA functionalized particles. Despite improvement on particle size (down to ca. 70 nm), the overall magnetization was rather low, at < 1.5 emu/g (compared to a typical value of 40–90 emu/g for magnetic nanoparticles), due to non-magnetic PGMA component. To simplify the material synthesis, preparation of NiFe_2_O_4_ nanoparticles in a two-step procedure was reported for immobilizing glucose dehydrogenase (GDH) directly from a cell lysate^31^. Unfortunately, this study lacks characterization data on the material for identifying the nature of the magnetic support being used. Other examples of magnetic carriers for enzyme immobilization can also be found in the literature^32,33^. Commercial magnetic carriers functionalized with Ni binding sites are also available for this purpose^34–36^ but they tend to be larger in particle size (250 nm–30 μm), which may reduce the binding capacity per mass of carrier.

**Figure 1:**
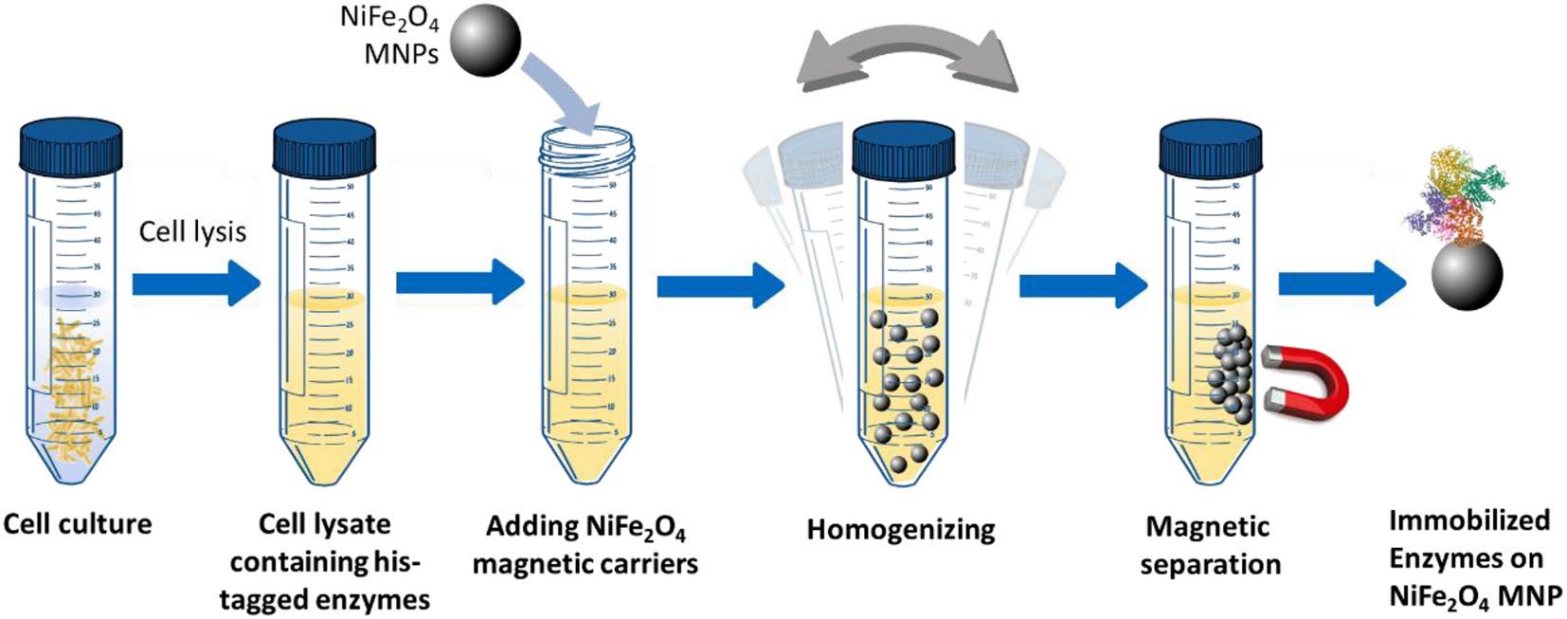
Schematic illustration for the direct purification/immobilization of enzymes from the cell lysate using NiFe_2_O_4_ MNPs.

In this work, we prepared NiFe_2_O_4_ MNPs via a one-step solvothermal synthesis and the nanoparticles were fully characterized using XRD, TEM, and VSM magnetometry. These MNPs were used for a one-pot purification and immobilization of his-tagged enzymes directly from cell lysates, with a simple protocol illustrated in Fig. 1. Firstly, GFP was used as a model protein to validate that immobilization via his-tag on NiFe_2_O_4_ MNPs occurred. Three enzymes, d-phenylglycine aminotransferase (d-PhgAT), *Halomonas elongata* ω-transaminase (HeωT), and *Bacillus subtilis* glucose dehydrogenase (*Bs*GDH), were then used as model enzyme molecules and their activity as the immobilized form was comparable to the immobilized enzymes prepared via a conventional two-step protocol (purification followed by immobilization). This is the first report presenting a protocol for direct enzyme immobilization from cell lysates using NiFe_2_O_4_ MNPs with no surface functionalization. This protocol significantly simplified the conventional enzyme immobilization procedure from purified enzymes. Moreover, the preparation procedure of the NiFe_2_O_4_ used is also considerably simpler than other surface functionalized magnetic nanoparticles, notably Ni–NTA functionalized Fe_3_O_4_ MNPs, which is commonly used for enzyme immobilization.

## Experimental

### Chemicals

Iron (III) chloride hexahydrate (FeCl_3_.6H_2_O, 98–102%), ethylene glycol (≥ 99%), urea (≥ 98%), polyvinylpyrrolidone (PVP) (MW 40,000), acetophenone (99%), sodium pyruvate (≥ 99%), (s)-methylbenzylamine (s-MBA) (≥ 98%), pyridoxal 5’-phosphate hydrate (PLP) (≥ 98%), nicotineamide adenine dinucleotide phosphate disodium salt (NADP^+^) (≥ 98%), nitrotetrazolium blue chloride (NBT), phenazine methosulfate (PMS) (90%), d-glucose (≥ 99.5%) yeast extract, tryptone digest, sodium chloride (≥ 99.5%), ampicillin sodium salt and phosphate buffered saline (PBS) were all purchased from Sigma Aldrich, UK. Nickel (II) chloride hexahydrate (NiCl_2_.6H_2_O, 97%), potassium dibasic monohydrogen phosphate (99% +) were supplied from Acros Organics, UK.

*Aequorea victoria* GFP his-tag (his-tagged green fluorescent protein) lyophilized (≥ 85%) was purchased from Thermo-Fisher Scientific, UK. Potassium monobasic dihydrogen phosphate anhydrous, dimethylsulfoxide (DMSO) (≥ 99.7%), sodium chloride (≥ 99.5%) and HEPES buffer were purchased from Fisher, UK. Imidazole was purchased from Fluorochem, UK.

NuPAGE™ 4–12% Bis–Tris Gel 1.0 mm × 10 well were purchased from Invitrogen. *E. coli* BL21 (DE3) competent cells were purchased from Agilent. *N*-cyclohexyl-3-aminopropanesulfonic acid (CAPS) was purchased from Fluorochem. All chemicals were used as received without further purifications. Procedure for growth and purification of *Halomonas elongata* ω-transaminase (HeωT), d-phenylglycine amino transferase (d-PhgAT), and *Bacillus subtilis* glucose dehydrogenase (*Bs*GDH) can be found in Supplementary Information SI.

### Preparation of nickel ferrite NiFe_2_O_4_ magnetic nanoparticles MNPs

Nickel ferrite magnetic nanoparticles (NiFe_2_O_4_ MNPs) was prepared using via solvothermal synthesis^37^. Nickel chloride hexahydrate (2.38 g) and iron chloride hexahydrate (5.41 g) were dissolved in 20 mL of ethylene glycol. A separate solution was prepared by dissolving urea (2.5 g) and polyvinylpyrrolidone PVP (0.2 g) with another 20 mL portion of ethylene glycol. These solutions were then fully mixed at room temperature until a homogenized solution was achieved. The solution was then transferred to a Teflon lined stainless steel autoclave (Parr acid digestion vessel, 45 mL) and heated in an oven to 180 °C for 20 h. The autoclave was then cooled to room temperature and the black NiFe_2_O_4_ MNPs were harvested with a magnet, and washed with distilled water for at least 20 times followed by ethanol a further 10 times. The NiFe_2_O_4_ MNPs were then dried at 60 °C under vacuum in a vacuum oven for 24 h.

### Characterization of NiFe_2_O_4_ MNPs

The as prepared NiFe_2_O_4_ MNPs were characterized using transmission electron microscopy TEM, SQUID (superconducting quantum interference device) magnetometry and powder x-ray diffraction XRD. The morphology of the NiFe_2_O_4_ MNPs was examined using a FEI TECNAI TF20 microscope fitted with a field emission gun and operated at 200 keV. The NiFe_2_O_4_ MNPs sample (< 0.1 mg) was suspended in ethanol using sonication and then deposited on a holey carbon (300 mesh) sample grid (Agar). The prepared sample grid was dried in air for at least 24 h before analysis. To study the magnetic properties of the NiFe_2_O_4_ MNPs, measurements of hysteresis loops were performed on a Quantum Design MPMS3 SQUID magnetometer equipped with a 7 T DC magnet. In a typical experiment, a sample was weighed into a gel capsule. A small drop of Eicosene (Sigma-Aldrich) preheated to 60 °C was added to the capsule to prevent the sample from moving within strong magnetic fields. The capsule then was weighed again to account for the mass of Eicosene. The hysteresis loops of the magnetic moment (M) versus field (H) for the samples were measured at room temperature with a maximum field of about ± 15 kOe. The crystal structure of NiFe_2_O_4_ MNPs was examined using a Panalytical diffractometer (Cu Kα radiation, λ = 1.5406 nm) for 2θ from 20°–80° at a scan rate of 2° per minute used to analyze the crystalline structure of the prepared NiFe_2_O_4_ sample.

### GFP binding 

To visualize the protein binding to NiFe_2_O_4_ MNPs surface, his-tagged green fluorescent protein (GFP) was used as the test protein molecule. NiFe_2_O_4_ MNPs (10 mg) were fully dispersed in 10 mL of 50 mM potassium phosphate buffer (pH 8) through sonication, then GFP at 0.025 μg/mL was added to the MNP suspension and left to interact for 4 h at 4 °C. To keep the fluorescence active, the suspension was wrapped in tin foil and kept in the dark for the duration of binding. After binding the supernatant was removed, the particles were washed until the supernatant appeared to show no fluorescence under a UV light. The GFP tagged MNPs (MNP-GFP) were then redispersed in 50 mM potassium phosphate buffer (pH 8) ready for fluorescence quantification. Fluorescence imaging was carried out using a fluorescent microscope (Zeiss AXIO Imager M2) under a × 10 magnification and blue light at 488 nm. The images from the sample were recorded using Zeiss AxioCam ERc 5 s in both brightfield mode and fluorescence mode.

### Protein expression and enzyme purification

All enzymes were prepared from recombinant protein (enzyme) expression in *E coli.* Bacteria^38,39^. In general, *E. coli* BL21 (DE3) cells were transformed via heat shock transformation with the desired plasmid. Selection was carried out on lysogeny broth (LB) agar supplemented with antibiotics. A starter culture of LB media was inoculated with a single colony. Starter culture was used to inoculate fresh LB media which was grown then induced using *iso*-propyl-β-d-1-thiogalactopyranoside (IPTG). The cells were harvested from the cultures expressing the protein before storing as a pellet at − 20 °C.

Cell pellets were re-suspended and lysed by sonication. Cell debris was removed and the remaining cell lysate was then filtered. The his-tagged enzymes were purified using IMAC using a HisTrap™ Nickel affinity column (Cytiva Lifesciences, UK), followed by SEC. In short, the binding and separation mechanism of his-tagged enzymes is based on the interaction between the his-tags and the Ni binding site on the stationary phase of the HisTrap™ column. Protein yield and concentration were determined and the purified protein was flash frozen and stored at − 80 °C. (See Sects. S1.1 and S1.2 in Supplementary Information for additional details on protein expression and purification.)

### Direct immobilization of d-PhgAT and HeωT from cell lysate 

In a typical experiment, 10 mg of NiFe_2_O_4_ MNPs are dispersed in 9 mL potassium phosphate buffer (50 mM, pH 8) with DMSO (0.25% v/v) and PLP (0.1 mM), and are sonicated for 15 min to ensure full dispersion of nanoparticles. Then 1 mL of cell lysate is added to the NiFe_2_O_4_ MNPs solution and rotated for 4 h at 4 °C. After immobilization the supernatant was removed, and the immobilized particles were washed with 5 portions of buffer (5 mL) to remove the undesirable components from the cell lysate. The immobilized enzyme on MNPs were then redispersed in 10 mL of 50 mM potassium phosphate buffer ready for reactions. These samples were denoted as MNP-d-PhgAT CL and MNP-HeωT CL, where “CL” indicates the immobilization was carried out directly from cell lysates. For each transaminase a further two purifications and immobilizations were carried out to also further asses if the nanoparticle concentration influenced the activity.

### Activity assay for d-PhgAT and HeωT 

The activity of both transaminases (d-PhgAT and HeωT) was determined by the rate of acetophenone production (at 245 nm) per minute by UV spectroscopy (Shimadzu UV–vis spectrophotometer, UVmini-1240). A typical reaction would take place at 37 °C and include a reaction mixture containing d-PhgAT or HeωT (0.018 mg/mL or 0.05 mg/mL respectively), sodium pyruvate (2.5 mM), (s)-methylbenzylamine (2.5 mM), PLP (0.1 mM) in potassium phosphate buffer (50 mM, pH8) and DMSO (0.25%v/v) in a final reaction volume of 1 mL^40,41^. Each assay was repeated at least three times and the specific activity was expressed as M/mg of purified enzyme.

### Purification and direct immobilization of BsGDH from cell lysate

The glucose dehydrogenase purification and immobilization from cell lysate was carried out using a similar protocol as immobilizing d-PhgAT and HeωT. In this immobilization, 10 mg of NiFe_2_O_4_ MNPs was dispersed fully in in 9 mL of potassium phosphate buffer via sonication. Then 1 mL of cell lysate was added to the reaction mixture and placed on an orbital mixer for 4 h at 4 °C. Once the purification/immobilization was complete, the supernatant was removed and the immobilized GDH particles (MNP-GDH CL) were washed with 5 volumes of 2 mL of buffer to remove any undesired components from the cell lysate. The immobilized enzymes were then redispersed in 10 mL of 50 mM potassium phosphate buffer (pH 8) ready to be used for assays. The volume of MNP-GDH CL was kept the same as the purified *Bs*GDH immobilization to be able to make a direct comparison to the purified enzyme.

### Activity assay for glucose dehydrogenase 

The activity of *Bs*GDH from *Bacillus subtilis* (0.041 mg/mL) was assessed at 580 nm using glucose (6 mM) and NADP^+^ (0.004 mM) as substrates, nitrotetrazolium blue chloride (NBT) (0.2 mg/mL) and phenazine methosulfate (PMS) (0.01 mg/mL) were added to the assay as they react with the NADPH produced by the GDH to produce an insoluble blue-purple formazan which can be measured at 580 nm^42^. The reaction was monitored in a Shimadzu UV–vis spectrophotometer (UVmini-1240).

## Results

### Characterizations of NiFe_2_O_4_ MNPs

**Figure 2:**
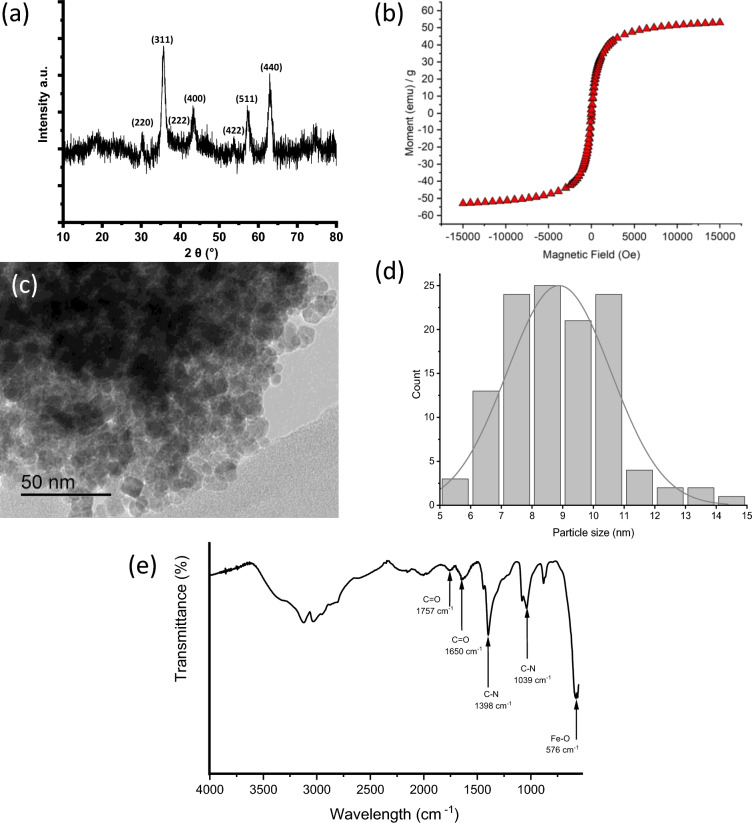
Characterization of NiFe_2_O_4_ magnetic nanoparticles MNPs. (**a**) XRD pattern for NiFe_2_O_4_ MNPs, (**b**) hysteresis loop of NiFe_2_O_4_ MNPs from SQUID measurement, (**c**) TEM of NiFe_2_O_4_ MNPs, (**d**) particle size distribution of NiFe_2_O_4_ MNPs, and (**e**) FTIR spectrum.

The prepared NiFe_2_O_4_ MNPs were characterized with TEM, VSM and XRD. The identity of the samples was confirmed to be NiFe_2_O_4_ by XRD (Fig. 2a) as the diffraction pattern was indexed and matched well with JCPDS card 74-2081. Using Scherrer analysis on the peaks, the size of the crystallites was found to be 10.6 ± 2.8 nm. The morphology of the NiFe_2_O_4_ sample was studied using TEM as shown in Fig. 2c, where a pseudo-spherical morphology is observed. The particle size of the NiFe_2_O_4_ sample was also analysed using ImageJ on 120 particles and the size distribution was shown in Fig. 2d. The average particle size was 8.9 ± 1.7 nm with the majority of particles (*ca.* 90%) were within the range of 6–11 nm. This result is consistent with the crystallite size estimated using Scherrer analysis, suggesting that most of these nanoparticles are of single domains. The magnetic property of the NiFe_2_O_4_ sample was analysed using SQUID magnetometry. Figure 2b shows the M v H curve with no hysteresis loop, suggesting that the sample is of superparamagnetic nature. This is common for magnetic nanoparticles of size smaller than 30 nm. The sample has shown a magnetization at saturation (*M*_*s*_) value 53.0 emu g^–1^ at H = 15 kOe, the value is comparable to those reported in the literature^37,42–44^. The FTIR spectrum of NiFe_2_O_4_ shown in Fig. 2e. The characteristic peak at 576 cm^–1^ was attributed to the Fe–O bond which is sometimes described as evidence of the formation of cubic spinel nickel ferrite structure. Other peaks that are present would be from the urea and PVP reactants. Characteristic peaks for urea at 1650 and 1039 cm^–1^ for C=O and C–N respectively, while PVP peaks appear at 1398 cm^–1^ for the aromatic amine (C-N) bond, and 1757 cm^–1^ for the C=O bond.

### Binding of his-tagged GFP

**Figure 3:**
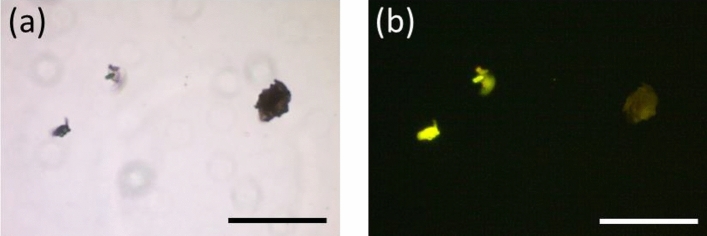
Fluorescence microscopy. (**a**) Brightfield and (**b**) fluorescence images of nickel ferrite MNP aggregates bound with his-tagged GFP. Scale bar = 100 μm.

In order to study binding his-tagged proteins with visual effect, his-tagged GFP was used. GFP is widely used as a probe for validating biological experiments using fluorescence microscopy, providing visual evidence^45^. In Fig. 3a and b, it can be seen that the NiFe_2_O_4_ MNP aggregates fluoresced under a blue light. This suggested that the his-tagged GFP has bound onto the NiFe_2_O_4_ MNPs directly.

### One-pot purification and immobilization for enzymes

**Figure 4:**
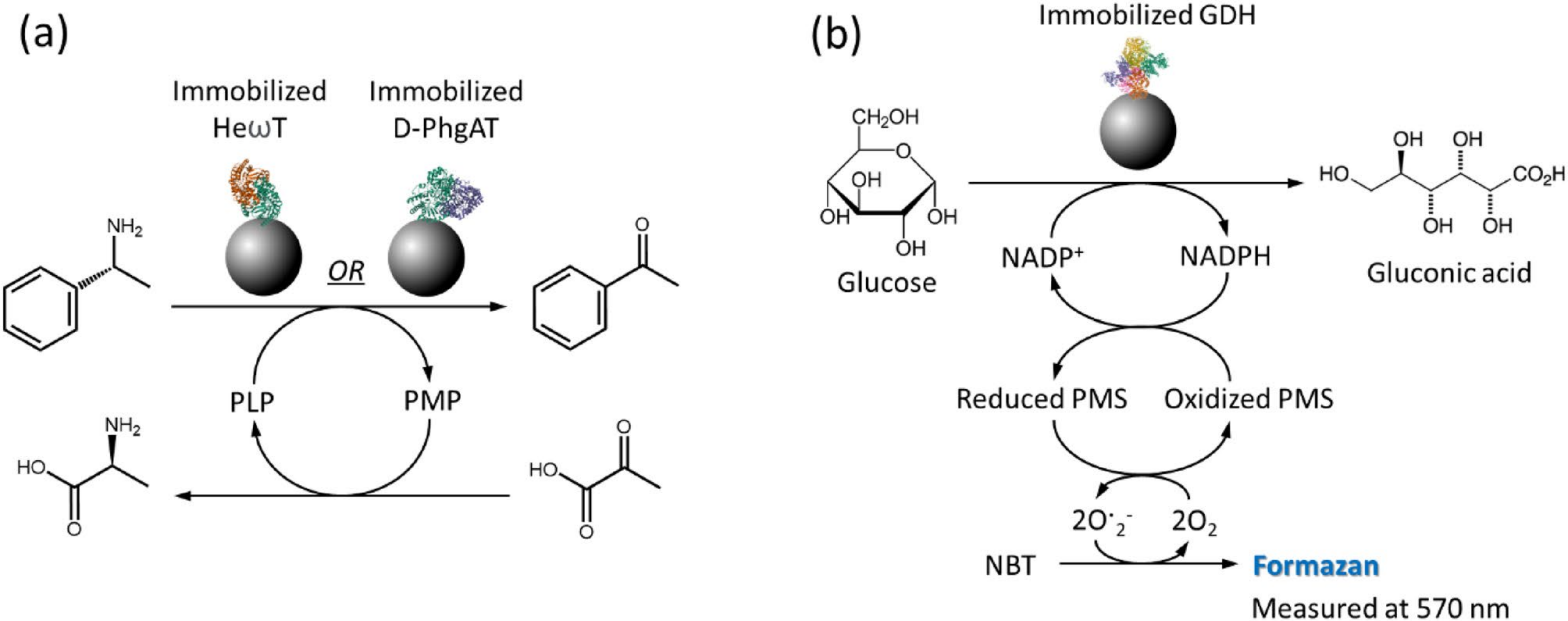
Schematic for the activity assays for (**a**) transaminases d-PhgAT and HeωT and (**b**) *Bs*GDH.

The goal of this study is to develop a fast, direct route to immobilize enzyme via his-tag binding. We have chosen three his-tagged enzymes for validating this method, d-PhgAT, HeωT and *Bs*GDH. The activities of these enzymes were compared in three different forms: free enzymes, immobilized enzymes from pre-purified form (the conventional route), and immobilized enzymes directly from cell lysates. Assay for both d-PhgAT and HeωT transaminases used the transamination reaction from s-methylbenzylamine to form glutamic acid, while the production of acetophenone was measured (Fig. 4a). For *Bs*GDH activity, an indirect method was adapted. The production of formazan was measured, following the scheme in Fig. 4b.

**Figure 5:**
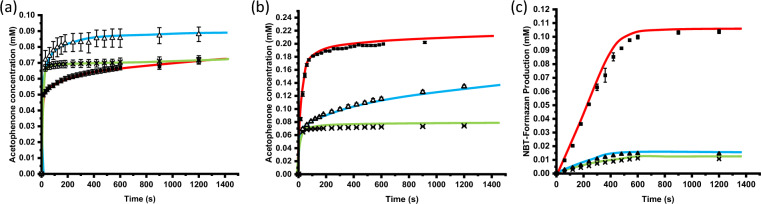
Enzymatic activities. Comparison plots for the activity assays for (**a**) d-PhgAT, (**b**) HeωT and (**c**) GDH. In all three plots, free enzymes were depicted as red lines with black squares, blue lines with hollow triangles for pre-purified enzymes immobilized on NiFe_2_O_4_ MNPs, and green lines with crosses for direct immobilization on NiFe_2_O_4_ MNPs from cell lysates without purification.

The initial activities (initial rates of reaction) and the activity curves of all three enzymes were shown in Table 1 and Fig. 5 respectively. In all three cases, initial activities of immobilized enzymes from the two methods (conventional route from pre-purified enzymes and direct immobilization from cell lysates) showed little difference, as shown in Table 1. This suggests that NiFe_2_O_4_ MNPs can be used as the carrier for direct enzyme immobilization from cell lysates without compromising on the enzyme activity. In case of d-PhgAT, both immobilized enzymes also showed an improvement on activity compared with the free enzyme. This was not the case in HeωT and *Bs*GDH where loss of activity was observable, which is commonly found in many immobilized enzyme systems. However, when comparing the activities among the immobilized enzymes, all three samples displayed little difference between two different immobilization methods, direct binding from cell lysates vs binding from pre-purified enzymes. This observation suggested that enzyme immobilization can be carried out without laborious pre-purification steps.

**Table 1:** Comparison of initial activity (initial rate) *based on a molar absorption coefficient of 12,300 for NBT-formazan^46^.

Activity (initial rate of reaction)	Free enzyme (nmol min^–1^)	Immobilization from pre-purified enzymes (nmol min^–1^)	Immobilization directly from cell lysates (nmol min^–1^)	Specific activity (unit) of free enzyme (U mg^–1^)
d-PhgAT	1.7	2.4	2.2	6.2
HeωT	4.1	2.4	2.1	35
*Bs*GDH*	0.21	0.03	0.02	251

Moreover, this direct immobilization method can also be operated in a larger scale. An experiment was set up with the undiluted cell lysate with d-PhgAT at 24 mL with a much higher concentration of NiFe_2_O_4_ MNPs (267 mg/mL, calculated based on the enzyme concentration in cell lysate). The initial activity of the immobilized enzyme was comparable to the smaller scale (1 mL) immobilization, as seen in Fig. S3. The recorded final conversion was lower possibly due to the lower immobilization efficiency using a higher MNP concentration. Nonetheless, this suggested that our system can possibly be scaled up, making it more suitable for use in industries.

## Discussion

**Figure 6:**
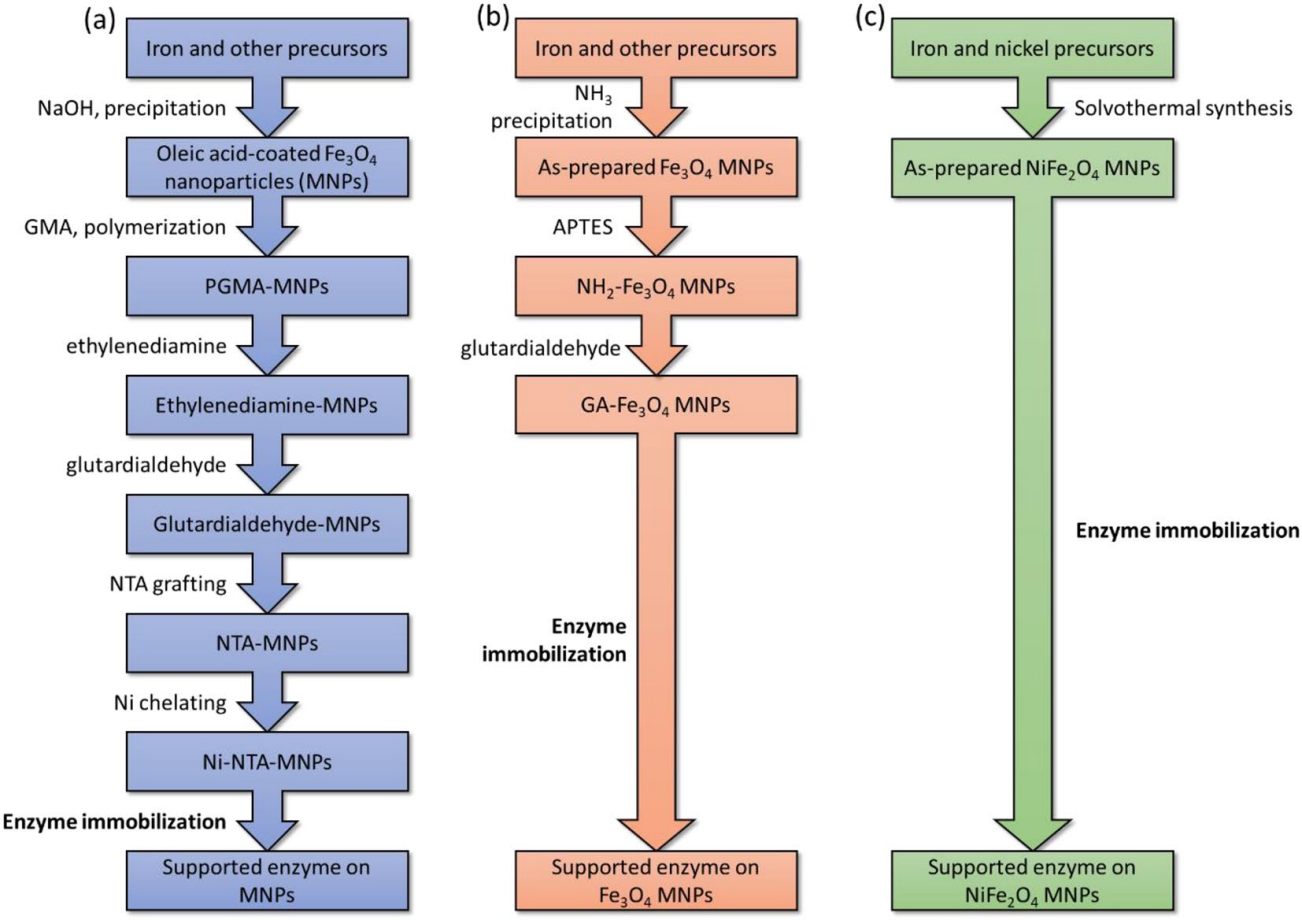
Comparison between different methods to prepare Ni-MNPs for protein binding and purification. Three synthesis procedures in the literature were compared based on the steps that are required to synthesize (**a**) Ni-NTA MNPs^40^ (**b**) glutardialdehyde MNPs (immobilization for non-his-tagged enzymes)^49^ and (**c**) NiFe_2_O_4_ MNPs used in the current study. No washing or separation step was included.

Protein purification and immobilization using his-tag technology is very useful as mentioned earlier. Using magnetic carriers has added an advantage of magnetic separation, which helps in recovering the desired products from a complex matrix, particularly where unwanted solid residues are in the matrix, including cell lysates^47^. However, most magnetic carrier materials reported in the literature for binding his-tagged proteins require multi-step protocols for their synthesis (see Fig. 6). For example, Zhou et al. reported a 6-step protocol to prepare Ni–NTA MNPs, which allows direct binding to his-tagged species (Fig. 6a)^28^. For immobilizing non-his-tagged proteins, glutardialdehyde-functionalized MNPs are commonly used but they still need a 3-step protocol for synthesis (Fig. 6b)^48^. Using glutardialdehyde functional groups for protein purification/immobilization possess a non-selective binding. This is likely to cause problem from cell lysates where many different protein types are present. Since NiFe_2_O_4_ MNPs have intrinsic Ni binding sites on surface, a one-step solvothermal synthesis can produce a ready-for-use carrier for binding his-tagged proteins (Fig. 6c). Generally, the more steps in synthesis, the protocol will incur a lower efficiency and a lower material yield, and also use more chemicals. Therefore, use of NiFe_2_O_4_ MNPs can also be seen as a greener option for purification and immobilization of his-tagged species by reducing use of unnecessary chemicals and emission of wastes. Under the current climate, such advantage can be particularly attractive in reducing the environmental impacts from biotechnological industries.

**Figure 7:**
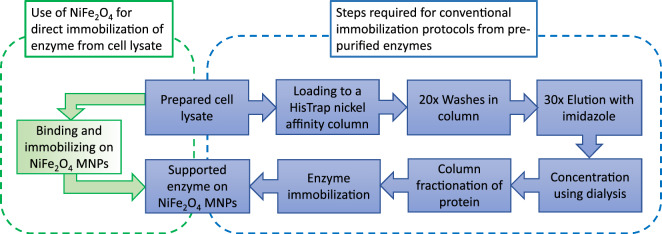
Comparison between direct enzyme immobilization to the conventional method. The following flow charts are to compare between direct enzyme immobilization from cell lysate using NiFe_2_O_4_ MNPs to the conventional immobilization methods from purified enzymes, which has 7 steps instead of 2 steps from direct immobilization demonstrated in this current work.

In addition to simplified synthesis protocol, purification with direct immobilization of his-tagged enzymes from cell lysates also help to improve the efficiency in protein/enzyme purification, in particular if the end use is as immobilized enzymes. Figure 7 illustrates the steps required for the immobilization of a purified his-tagged enzyme from cell lysate in a conventional protocol, compared to a one-step direct immobilization demonstrated in our work. The key feature highlighted here is that little loss in activity was shown from three different enzymes comparing to those from conventional immobilization using pre-purified enzymes, suggesting that direct immobilization can also simplify the immobilization protocol. Again, similar to materials synthesis, the fewer steps required for immobilization also result in reducing use of unnecessary chemical and waste emission, making the method even greener.

## Conclusion

We have demonstrated a solvothermal synthesis method for NiFe_2_O_4_ MNPs. From the TEM analysis and VSM measurement, these NiFe_2_O_4_ MNPs have an average diameter of 8.9 ± 1.7 nm and a magnetization at saturation of 59 emu g^–1^. These MNPs were proven to bind his-tagged GFP as observed using fluorescent microscopy. For direct enzyme immobilization from cell lysates, all three enzymes (d-PhgAT, HeωT and BsGDH) showed activities (initial rates) comparable to the correspondent immobilized enzymes prepared via a conventional method (i.e. purification followed by immobilization). More interestingly, immobilized d-PhgAT samples, via either direct immobilization from cell lysate or conventional immobilization method, showed a higher activity than the purified free enzymes, at only 1.7 nmol min^–1^ compared with 2.2 and 2.4 nmol min^–1^ recorded from the immobilized samples.

Regarding synthesis of carriers, the one-step protocol for NiFe_2_O_4_ MNPs is a significant advancement compared to other magnetic nano-carriers with Ni sites, that could take as many as 6 steps to prepare. Since many engineered enzymes were modified to tether a polyhistidine chain for purification purpose, direct binding using this functionality onto a nickel-based carrier would save the use of binding agents (e.g. EDC/NHS) that may also alter the chemical structure of enzymes due to crosslinking. Exploiting the Ni-histidine affinity also offers a high selectivity/specificity for the desired proteins. Using direct immobilization method from cell lysates also reduce time, manpower and chemicals during purification of enzymes. Therefore, this simple, direct immobilization of enzymes using NiFe_2_O_4_ MNPs can enhanced the sustainability of relevant pharmaceutical and chemical industries. Furthermore, enzymatic reactions can be considered to be greener due to their low-energy requirement and solvent-free nature. Reuse and recycling of enzymes using magnetic separation can make many enzymatic reactions feasible for large-scale applications in industries by reducing cost and environmental impact. 

### Supplementary Information

Supplementary Information.

## Data Availability

All data generated and analyzed during this research are included in this published article and additional file.
