# Supplementary material for: Direct purification and immobilization of his-tagged enzymes using unmodified nickel ferrite NiFe2O4 magnetic nanoparticles
Source: Sci Rep. 2023 Dec 6;13:21549. doi: 10.1038/s41598-023-48795-x (PMC10700653; doi:10.1038/s41598-023-48795-x)
Supplement: Supplementary file 1 — Supplementary Information. [file 41598_2023_48795_MOESM1_ESM.docx]

**Supporting Information**

**Direct purification and immobilization of his-tagged enzymes using unmodified nickel ferrite NiFe_2_O_4_ magnetic nanoparticles**

Elizabeth C.H.T. Lau^a^, Kimberley C. Dodds^b^, Catherine McKenna^b^, Rhona M. Cowan^b^, Alexey Y. Ganin^c^, Dominic J. Campopiano^b^, Humphrey H.P. Yiu^a,^*

^a^ Chemical Engineering, School of Engineering and Physical Sciences, Heriot-Watt University, Edinburgh, EH14 4AS, U.K.

^b^ School of Chemistry, University of Edinburgh, Edinburgh, EH9 3FJ, U.K.

^c^ School of Chemistry, University of Glasgow, Glasgow, G12 8QQ, U.K.

**S1. Materials and methods**

Additional details for enzyme expression and purification procedures

S1.1 Enzyme Expression

All cell cultures were grown in the presence of ampicillin (100 µg/mL). Plasmids (2 µL) containing gene sequences of HeωT, d-PhgAT or *Bs*GDH were inserted into *E. coli* BL21 (DE3) chemically competent cells (10 µL) *via* heat shock transformation and selection was carried out on lysogeny broth (LB) agar. A starter culture of LB media (250 mL) was inoculated with a single colony and grown at 37 °C, 200 rpm overnight. Then fresh LB media (1 L) was inoculated with the previous starter culture to an OD_600_ of 0.1. The cells were grown at 37 °C, 200 rpm until and OD_600_ of 0.6-0.8 was reached. Protein expression was induced with *iso*-propyl-β-D-1-thiogalactopyranoside (IPTG) (HeωT - 1 mM, d-PhgAT/ *Bs*GDH – 0.1 mM) and the temperature was lowered ≤20 °C (HeωT / d-PhgAT - 20 °C, *Bs*GDH – 16 °C), 200 rpm overnight. The cells were harvested by centrifugation (4000 x g, 1 h, 4 °C). The cell pellets were resuspended in phosphate buffer saline (PBS), and re-collected by centrifugation (5000 x g, 30 min, 4 °C) before storing at -20 °C.

S1.2 Enzyme Purification

Cell pellets were re-suspended in the appropriate binding buffer (buffers A,D or G; Table S1) and lysed by sonication (30s on, 30s off, 15 cycles). The sonicated cells were then centrifuged (9500 x g, 45 min, 4 °C) to remove cell debris from the lysate. Cell lysate was then filtered through Millex HA filters (0.45 µM) and loaded onto pre-equilibrated His-Trap Nickel affinity column with binding buffer using an ÄKTA explorer (Cytiva Lifesciences, UK). The column was washed with 10 column volumes of binding buffer. Elution buffer (buffers B, E or H; Table S1) was applied with a gradient 0 to 100 % over 30 column volumes. Fractions collected were concentrated to <1 mL by vivaspin 20 molecular weight cut off (MWCO) (HeωT /d-PhgAT- 30 kDa, *Bs*GDH- 5 kDa satorius). The concentrated protein sample was loaded onto a Hi Prep^TM^ 16/600 superdex^TM^ S-200 size exclusion column (SEC) (120 mL) pre-equilibrated with gel filtration buffer (buffers C, F or I; Table S1). Elution of the recombinant protein proceeded at flow rate 0.5 mL/min. Protein concentration was determined by nano drop (see table S2 for details). Purified protein was flash frozen with liquid nitrogen and stored at -80 °C.

| **Enzyme** | **Buffer Reference** | **Buffer** | **Contents** |
| --- | --- | --- | --- |
| **HeωT** | A | Binding | KPhos (50 mM, pH 8), NaCl (100 mM), PLP (0.1 mM) and imidazole (30 mM) |
|  | B | Elution | KPhos (50 mM, pH 8), NaCl (100 mM), PLP (0.1 mM), imidazole (300 mM) |
|  | C | Gel Filtration | KPhos (50 mM, pH 8), NaCl (150 mM), PLP (0.1 mM) |
| **d-PhgAT** | D | Binding | CAPS (0.1 M, pH 9), NaCl (150 mM), imidazole (20 mM), PLP (0.2 mM). |
|  | E | Elution | CAPS (0.1 M, pH 9), NaCl (150 mM), imidazole (500 mM), PLP (0.1 mM). |
|  | F | Gel Filtration | CAPS (0.1 M, pH 9), NaCl (150 mM), PLP (0.1 mM). |
| ***Bs*GDH** | G | Binding | HEPES (20 mM, pH 7.5), NaCl (500 mM), imidazole (20 mM) |
|  | H | Elution | HEPES (20 mM, pH 7.5), NaCl (500 mM), imidazole (500 mM) |
|  | I | Gel Filtration | HEPES (20 mM, pH 7.5) NaCl (500 mM) |

**Table S1.** Table summarising various buffers used for the purification of enzymes HeωT, d-PhgAT and *Bs*GDH

**Table S2:** Data on recombinant enzymes

| **Enzyme** | **Uniprot code** | **Molecular Weight (Monomer kDa)** | **Extinction coefficient, ε (M^-1^ cm^-1^ at 280 nm)^i^** | **Average Yield of culture / g of wet cell mass** |
| --- | --- | --- | --- | --- |
| **HeωT** | E1V9I3 | 54 | 60850 | 11.3 mg |
| **d-PhgAT** | Q6VY99 | 49 | 37390 | 20.0 mg |
| ***Bs*GDH** | P12310 | 30 | 29910 | 11.25 mg |

i – ε was determined by the online Protparam tool.


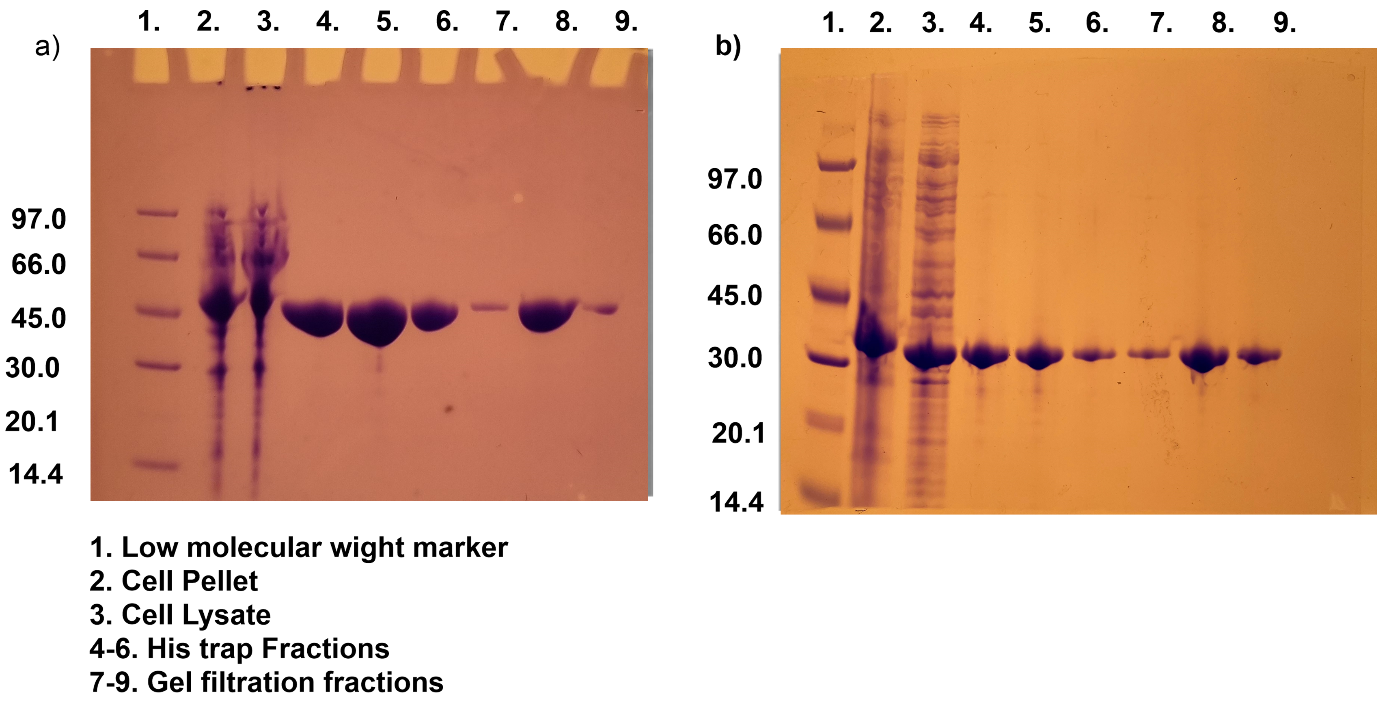


**Figure S1** SDS-PAGE gels from purification of a) d-PhgAT, b) *Bs*GDH


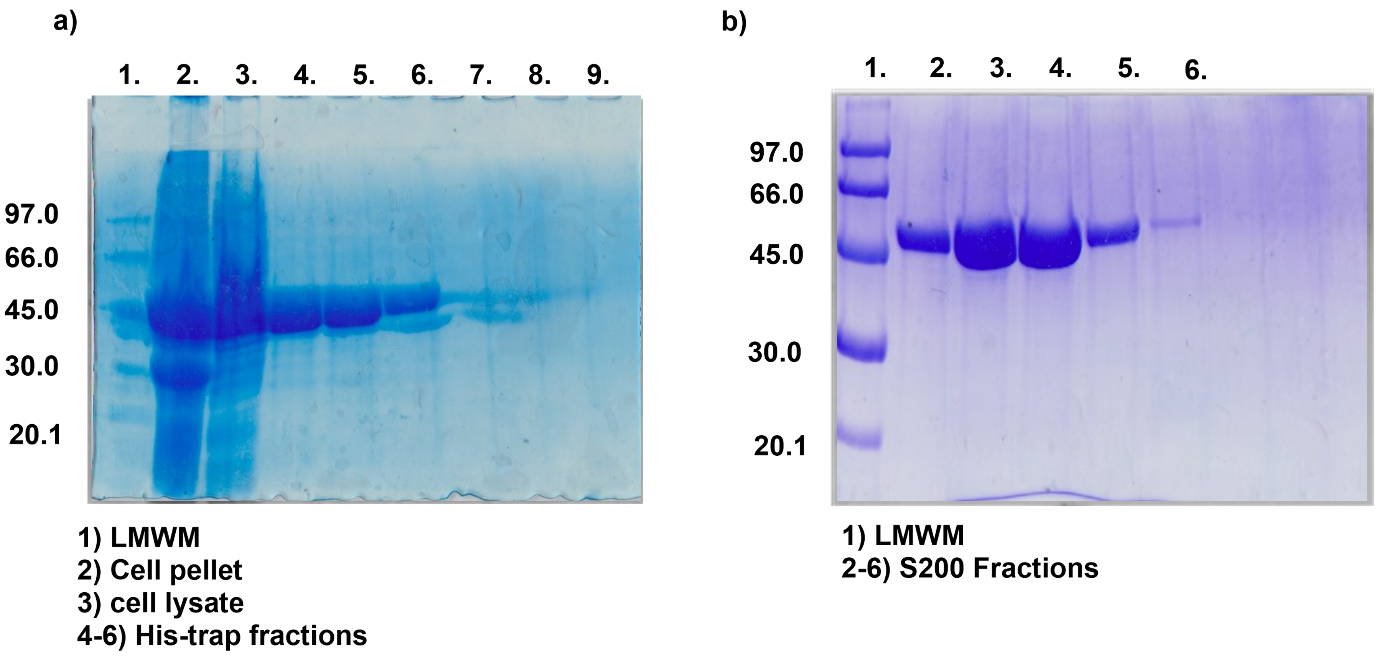


**Figure S2** SDS-PAGE gels from purification of HeωT a) Cell pellet, cell lysate and IMAC fractions, b) S200 SEC fractions.

**Figure S3** Comparison on activity between D-PhgAT immobilized on a small (1 mL) scale (in blue) and immobilized on a large scale without dilution (24 mL, in purple).
